# Associations of self-reported physical activity and anxiety symptoms and status among 7,874 Irish adults across harmonised datasets: a DEDIPAC-study

**DOI:** 10.1186/s12889-020-08481-3

**Published:** 2020-03-20

**Authors:** Cillian P. Mc Dowell, Angela Carlin, Laura Capranica, Christina Dillon, Janas M. Harrington, Jeroen Lakerveld, Anne Loyen, Fiona Chun Man Ling, Johannes Brug, Ciaran MacDonncha, Matthew P. Herring

**Affiliations:** 1 The Irish Longitudinal Study on Ageing (TILDA), Lincoln Gate, Trinity College Dublin, Dublin 2, Ireland; 2 Department of Physical Education and Sport Sciences, University of Limerick, Limerick, Ireland; 3 Health Research Institute, University of Limerick, Limerick, Ireland; 4 Centre for Exercise Medicine, Physical Activity and Health, Sport and Exercise Sciences Research Institute, Ulster University, Northern Ireland, UK; 5 Department of Movement, Human and Health Sciences, University of Rome Foro Italico, Rome, Italy; 6 School of Public Health, University College Cork, Cork, Ireland; 7 Department of Epidemiology and Biostatistics, Amsterdam UMC, VU University Medical Center, Amsterdam Public Health Research Institute, Amsterdam, The Netherlands; 8 Julius Center for Health Sciences and Primary Care, University Medical Center Utrecht, Utrecht, the Netherlands; 9 Global Geo Health Data Center, Utrecht University, Utrecht, The Netherlands; 10 Centre for Nutrition, Prevention and Health Services, National Institute for Public Health and the Environment, Bilthoven, the Netherlands; 11 Department of Sport, Exercise & Rehabilitation, Northumbria University, Newcastle upon Tyne, UK; 12 Amsterdam School for Communication Research, University of Amsterdam, Amsterdam, the Netherlands; 13 National Institute for Public Health and the Environment, Bilthoven, the Netherlands

**Keywords:** Physical activity, Mental health, Elderly, Ireland, Cross-sectional

## Abstract

**Background:**

Anxiety is an adaptive response to an objective or perceived threat; however, when symptoms become severe and chronic it that can become a maladaptive anxiety disorder. Limited evidence suggests that physical activity may be associated with prevention against anxiety. This study uses data from The Irish Longitudinal Study on Ageing (TILDA) and The Mitchelstown Cohort Study to investigate cross-sectional associations between physical activity and anxiety symptoms and status among Irish adults.

**Methods:**

Both datasets were harmonized (*n* = 7874). The short form International Physical Activity Questionnaire measured physical activity. Participants were classified as meeting World Health Organization physical activity guidelines (≥150 min weekly of moderate intensity physical activity, ≥75 min weekly of vigorous intensity physical activity, or ≥ 600 MET-minutes) or not. They were also divided into three groups based on weekly MET-minutes of moderate-to-vigorous physical activity (Low: 0–599; Moderate: 600–1199; High: ≥1200), and three groups based on weekly minutes of walking (Low: 0–209; Moderate: 210–419; High: 420+). Anxiety symptoms were measured by the Hospital Anxiety and Depression Scale with a score of ≥8 indicating anxiety. Binomial logistic regression, adjusted for relevant confounders examined physical activity–anxiety associations.

**Results:**

Females had higher rates of anxiety than males (28.0% vs 20.0%; *p* < 0.001). Following adjustment for relevant covariates, meeting physical activity guidelines was associated with 13.5% (95% CI: 2.0–23.7; *p* = 0.023) lower odds of anxiety. Moderate and High physical activity were associated with 13.5% (− 11.0–32.6; *p* = 0.254) and 13.6% (1.4–4.2; *p* = 0.030) lower odds of anxiety compared to Low physical activity, respectively. Moderate and High walking were associated with 2.1% (− 14.5–16.3; *p* = 0.789) and 5.1% (− 9.3–17.6; *p* = 0.467) lower odds of anxiety compared to Low walking, respectively.

**Conclusion:**

Meeting physical activity guidelines is associated with lower odds of anxiety, but the strength of associations did not increase considerably with increased physical activity levels.

## Introduction

Anxiety is an adaptive response to an objective or perceived threat; however, when symptoms become severe and chronic it can develop into a maladaptive anxiety disorder [1]. As the most prevalent class of mental disorders [2], anxiety disorders can have a substantial personal and economic cost [3]. They are the sixth leading cause of years lived with disability [2], and are more disabling than many medical conditions, including arthritis, asthma, back/neck pain, and cancer, predominantly within social life and personal relationship domains [4]. Anxiety symptoms and disorders are also associated with cardiovascular disease and associated premature mortality and various other comorbid medical conditions [5, 6]. Due largely to increased disability, anxiety disorders also have a large economic burden [7]; in 2010 they had an estimated cost of over €74 billion in Europe [3]. Although the economic burden is lower compared to €126 billion for cancer in 2009 [8] and €111 billion for cardiovascular disease in 2015 [9], this still represents a substantial burden. Moreover, it is plausible that this burden is even greater in Ireland given its economic difficulties in 2007 and the large body of literature demonstrating a possible association between economic recession and mental health [10].

Among individuals with anxiety, numbers seeking care can be low [11], as anxiety is can be interpreted as a normal, healthy response to adversity [12]. Among those who do seek treatment, common frontline treatments include serotonin reuptake inhibitors [13] which can be expensive and have negative side-effects [14] and cognitive behavioural therapy [15] which is only moderately effective [16]. Thus, there is continued interest in investigating potential cost-effective and accessible prevention, self-management, and treatment strategies for anxiety symptoms and disorders.

One strategy may be physical activity. Randomised controlled trials have demonstrated the benefits of exercise, a sub-category of physical activity performed to improve physical fitness, for anxiety symptoms [17, 18], generalized anxiety disorder (GAD) [19], obsessive-compulsive disorder [20], and panic disorder [21], and among a diverse range of populations. Moreover, the available evidence supports the biological plausibility of physical activity effects on anxiety. For example, there is growing evidence that exercise can improve hippocampal functioning [22] and volume [23], potentially improving decreased hippocampal volumes displayed by people with GAD [24] and social phobia [25]. Additionally, inflammation and nitrogen and oxidative stress may be important factors in the development of anxiety disorders as they can subsequently alter neuroplasticity, neurogenesis, and neurotrophins, while these same pathways are influenced by physical activity [26]. Despite this, few epidemiological studies have examined physical activity associations with anxiety relative to other mental health disorders such as depression [27]. Some evidence exists supporting cross-sectional, inverse association of physical activity with anxiety symptoms [28] and disorders [29]. The World Health Organization (WHO) recommends engaging in ≥150 min of weekly moderate intensity physical activity, ≥75 min weekly of vigorous intensity physical activity, or an equivalent combination of weekly physical activity for health benefits [30]; however, epidemiological studies examining associations of meeting the physical activity guidelines with anxiety are sparse [31–35]. Further, the WHO recommends doubling this dose (i.e., ≥300 min of moderate, ≥150 min of vigorous, or an equivalent combination) for additional health benefits. Given the worldwide promotion of the benefits of meeting these guidelines, it is important to clarify their association with anxiety symptoms and status. Furthermore, walking is an accessible behaviour for all ages and sexes that is known to confer many physical health benefits; however, just one longitudinal [36] and few cross-sectional [37] studies have examined associations of walking with anxiety.

Conducting large-scale research projects can have a substantial cost (e.g., both financial and time), so the utilisation of existing data by data sharing, dataset pooling, and harmonisation of relevant variables may be extremely beneficial [38–40]. Benefits include improving the cost-effectiveness of research and increasing the possibility for analyses on subsamples and statistical power [41, 42]. Thus, the current study harmonised two existing datasets to examine cross-sectional associations of walking and moderate-to-vigorous physical activity with anxiety symptoms and status. The authors hypothesised that anxiety would be inversely associated with physical activity and walking and that stronger associations would be observed with increased doses of walking and physical activity.

## Methods

This study adhered to Strengthening the Reporting of Observational Studies in Epidemiology (STROBE) guidelines [43].

### Participating studies

The selection of participating studies has been described elsewhere [44]. Briefly, the platform for the current research was the DEterminants of DIet and Physical ACtivity Knowledge Hub (DEDIPAC-KH) [45, 46]. Within DEDIPAC, accessible cross-sectional datasets with relevant data on physical activity and anxiety were identified in a large compendium for harmonisation [47]. Two datasets, The Irish Longitudinal Study on Ageing (TILDA) and The Mitchelstown Cohort Study were harmonised for the present study.

The Irish Longitudinal Study on Ageing (TILDA) is a study of 8175 community-dwelling adults aged ≥50 years and their partners of any age, living in the Republic of Ireland [48]. For the current study, Wave One (2009–2011) data were used. Data for 1804 participants aged 55–74 year from the second phase of the Cork and Kerry Diabetes and Heart Disease (2010) Study were provided by the Mitchelstown Cohort Study [49]. This study was designed to provide a profile of glucose tolerance status, cardiovascular health, and their related factors in an Irish adult general population sample. A six-person expert consensus group agreed appropriate variable harmonisation methods for physical activity, anxiety, participant age, sex, body mass index (BMI), smoking status, marital status, and education.

### Physical activity

The short-form International Physical Activity Questionnaire (IPAQ-SF) measured physical activity [50, 51]. Participants reported the number of days and duration of vigorous intensity, moderate intensity, and walking activities undertaken during the previous 7 days and were classified according to whether or not they met WHO physical activity guidelines [30]. Based on weekly metabolic equivalent,minutes (MET-mins) of moderate and vigorous physical activity, participants were divided into Low (0–599 MET^.^mins), Moderate (600–1199 MET^.^mins), and High (≥1200 MET^.^mins) dose categories [30]. Additionally, respondents were categorized as Low walkers if they reported walking < 210 min^.^week^− 1^ (i.e., < 30 min^.^day^− 1^), Moderate walkers if they reported walking 210–419 min^.^week^− 1^ (i.e., 30–59 min^.^day^− 1^), and High walkers if they walked ≥420 min^.^week^− 1^ (i.e., ≥60 min^.^day^− 1^).

### Anxiety

Anxiety was assessed by the Hospital Anxiety and Depression Scale anxiety subscale (HADS-A) [52] which has been validated in a random sample of adults aged ≥66 years in the Netherlands (Cronbach’s α = 0.82) [53]. Probable anxiety was indicated by scores ≥8. This cut-off has been demonstrated to have good specificity and sensitivity [54].

### Covariates

Covariates were selected based on known associations with physical activity or anxiety [34, 45, 55–57]. These included age (50–59, 60–69, 70–79, and 80+ years), sex (male or female), and BMI categorized as using established WHO thresholds (i.e., underweight: < 18.5 kg.m^− 2^; normal weight: 18.5–24.99 kg.m^− 2^; overweight: 25–29.99 kg.m^− 2^; obese: ≥30 kg.m^− 2^) [58]. Smoking status (never, former, or current smoker), marital status (married/living with a partner as if married, never married, separated, divorced, or widowed), and highest level of education achieved (none/primary, secondary level, third level or higher) were also assessed.

### Analyses

Similar to previous analyses [44] Chi-square tests examined differences between datasets in proportions of probable anxiety status, age categories, sex, BMI categories, smoking status, education, marital status, and physical activity. To determine the source of statistically significant Chi-square tests *Z* tests were calculated for column proportions for each row in the Chi-square contingency table and Bonferroni adjusted [59]. Binomial logistic regression quantified crude and adjusted associations (i.e., odds ratios (ORs) and 95% confidence intervals (95%CIs)) between physical activity and anxiety, within each dataset. Covariates in adjusted models were age, sex, BMI, smoking status, education, and, marital status.

Within the integrated dataset, Chi-square tests examined differences in elevated anxiety symptom status, ten-year age categories, sex, BMI categories, smoking status, education, and marital status between individuals meeting and not meeting physical activity guidelines. To determine the source of statistically significant Chi-square tests *Z* tests were calculated for column proportions for each row in the Chi-square contingency table and Bonferroni adjusted [59]. Binomial logistic regression quantified crude and adjusted associations between physical activity and anxiety. Covariates in adjusted models were age, sex, BMI, smoking status, education, marital status, and dataset. Likelihood ratio tests examined covariate significance in total population analyses in the integrated dataset. Additionally, a separate test of the model was run including the interaction between physical activity and sex. Differences in continuous anxiety symptoms between those meeting and not meeting physical activity guidelines, physical activity dose groups, and sexes were quantified by one-way ANOVAs followed by Bonferroni-corrected *post-hoc* tests. The magnitude of differences in anxiety symptom scores between meeting physical activity guidelines, physical activity dose categories, and sexes were quantified by Hedges’ *g* effect sizes and associated 95%CIs [60]. Effect sizes of 0.2, 0.5, and 0.8 were considered small, medium, and large, respectively [61].

## Results

### Dataset characteristics

Harmonised data characteristics are presented in Table 1. Results from Chi-square tests and follow-up *Z* tests are shown in Table 2.

**Table 1:** Study characteristics

	TILDA (n (%))	Mitchelstown (n (%))	***P***-value
**Age (years)**			
< 50	267 (4.2)_a_	2 (0.1)_b_	**< 0.001**
50–59	2571 (40.4)_a_	852 (56.9)_b_	
60–69	1995 (31.3)_a_	621 (41.5)_b_	
70–79	1162 (18.2)_a_	23 (1.5)_b_	
80+	374 (5.9)_a_	0 (0.0)_b_	
**Sex**			
Male	2779 (43.6)_a_	720 (48.1)_b_	**0.002**
Female	3597 (56.4)_a_	778 (51.9)_b_	
**Body Mass Index**			
Underweight	26 (0.5)	6 (0.4)	0.281
Normal	1131 (22.5)	354 (23.7)	
Overweight	2166 (43.1)	667 (44.6)	
Obese	1700 (33.8)	468 (31.3)	
**Education**			
Primary	1611 (26.9)_a_	351 (24.7)_a_	**0.002**
Secondary	2669 (44.6)_a_	706 (49.8)_b_	
Tertiary	1700 (28.4)_a_	362 (25.5)_b_	
**Marital status**			
Married/ living with a partner as if married	4676 (73.3)_a_	1180 (78.9)_b_	**< 0.001**
Single (never married)	541 (8.5)_a_	129 (8.6)_a_	
Separated	250 (3.9)_a_	64 (4.3)_a_	
Divorced	145 (2.3)_a_	33 (2.2)_a_	
Widowed	764 (12.0)_a_	89 (6.0)_b_	
**Smoker**			
Never	2820 (44.2)_a_	726 (49.8)_b_	**< 0.001**
Past	2457 (38.5)_a_	517 (35.4)_b_	
Current	1099 (17.2)_a_	216 (14.8)_b_	
**Meeting PA Guidelines**			
Yes	3057 (47.9)_a_	460 (30.7)_b_	**< 0.001**
No	3319 (52.1)_a_	1038 (69.3)_b_	
**PA Categories**			
Low	3319 (52.1)_a_	1038 (69.3)_b_	**< 0.001**
Moderate	440 (6.9)_a_	87 (5.8)_a_	
High	2617 (41.0)_a_	373 (24.9)_b_	
**Walking categories**			
Low	2839 (44.5)_a_	914 (61.1)_b_	**< 0.001**
Moderate	1394 (21.9)_a_	281 (18.8)_b_	
High	2143 (33.6)_a_	301 (20.1)_b_	

Different subscript letters indicate a subset of each category whose column proportions differ statistically significantly at the 0.05 level

**Table 2:** Participant characteristics

	HADS-A < 8 (n (%))	HADS-A ≥ 8 (n (%))	***P***-value
**Meeting PA Guidelines**			
Yes	2696 (45.3)_a_	802 (42.6)_b_	**0.038**
No	3252 (54.7)_a_	1105 (57.4)_b_	
**PA categories**			
Low	3252 (54.7)	1105 (57.4)	0.087
Moderate	411 (6.9)	116 (6.0)	
High	2285 (38.4)	705 (36.6)	
**Walking categories**			
Low	2831 (47.6)	922 (47.9)	0.815
Moderate	1259 (21.2)	416 (21.6)	
High	1857 (31.2)	587 (30.5)	
**Age**			
< 50	179 (3.0)_a_	90 (4.7)_b_	**< 0.001**
50–59	2436 (41.0)_a_	987 (51.3)_b_	
60–69	2062 (34.7)_a_	554 (28.8)_b_	
70–79	954 (16.1)_a_	231 (12.0)_b_	
80+	312 (5.2)_a_	62 (3.2)_b_	
**Sex**			
Male	2798 (47.0)_a_	701 (36.4)_b_	**< 0.001**
Female	3150 (53.0)_a_	1225 (63.6)_b_	
**Body Mass Index**			
Underweight	20 (0.5)_a_	33 (0.7)_a_	**0.011**
Normal	990 (25.8)_a_	1160 (24.9)_a_	
Overweight	1708 (44.4)_a_	1883 (40.4)_b_	
Obese	1126 (29.3)_a_	1580 (33.9)_b_	
**Education**			
Primary	1453 (26.1)	509 (27.9)	0.293
Secondary	2555 (45.8)	820 (45.0)	
Tertiary	1567 (28.1)	495 (27.1)	
**Marital status**			
Married/ living with a partner as if married	4443 (74.7)_a_	1413 (73.4)_a_	**0.013**
Single (never married)	509 (8.6)_a_	161 (8.4)_a_	
Separated	223 (3.8)_a_	91 (4.7)_a_	
Divorced	118 (2.0)_a_	60 (3.1)_b_	
Widowed	653 (11.0)_a_	200 (10.4)_a_	
**Smoker**			
Never	2748 (46.5)_a_	798 (41.5)_b_	**< 0.001**
Past	2282 (38.6)_a_	692 (36.0)_b_	
Current	883 (14.9)_a_	432 (22.5)_b_	

Different subscript letters indicate a subset of each category whose column proportions differ statistically significantly at the .05 level

### Participant characteristics

A total of 7874 and 6059 respondents were included in crude and adjusted analyses, respectively. Participant characteristics by anxiety status in the integrated dataset are presented in Table 2. The overall prevalence of anxiety was 24.5%. Females were more likely to report anxiety (χ^2^ (1, *N* = 7874) = 66.76, *p* < 0.001) than males (28.0% vs 20.0%) and reported significantly higher anxiety symptoms (5.93 ± 3.55) than men (5.15 ± 3.24; F_(1,7872)_ = 101.73, *p* < 0.001; *g* = 0.23; 95%CI: 0.18 to 0.27). Meeting guidelines (χ^2^ (1, *N* = 7874) = 4.29, *p* = 0.038), age (χ^2^ (4, *N* = 7867) = 90.89, *p* < 0.001), BMI (χ^2^ (3, *N* = 6518) = 11.22, *p* = 0.011), marital status (χ^2^ (4, *N* = 7871) = 12.60, *p* = 0.013), and smoking status (χ^2^ (2, *N* = 7835) = 59.61, *p* < 0.001) significantly differed according to anxiety status. Results from follow-up *Z* tests are shown in Table 1. The proportions of people who met physical activity guidelines (χ^2^ (1, *N* = 7874) = 0.91, *p* = 0.34) and who had anxiety (χ^2^ (1, *N* = 7874) = 0.02, *p* = 0.90) did not differ between those who were included in crude analyses but excluded from adjusted analyses due to missing covariate information.

### Integrated dataset analyses

Results for logistic regression models run within each dataset are reported in Table 3. Integrated dataset analyses are reported in Table 3. Following full adjustment, meeting physical activity guidelines was associated with 13.5% (95% CI: 2.0 to 23.7; *p* = 0.023) lower odds of anxiety. Age, sex, education, and smoking status were significant covariates (all *p* ≤ 0.001). Compared to those aged less than 50 years, those aged 50–59 (OR = 0.957, 95%CI = 0.703–1.303; *p* = 0.782), 60–69 (0.617, 0.449–0.847; *p* = 0.003), 70–79 (0.470, 0.331–0.667; *p* < 0.001), and 80+ (0.359, 0.222–0.581; *p* < 0.001) were less likely to report anxiety. Compared to females, males (0.672, 0.590–0.795; *p* < 0.001) were less likely to report anxiety. Compared to those only educated to primary level, those educated to secondary (0.780, 0.668–0.910; *p* = 0.002) and tertiary (0.745, 0.628–0.883; *p* = 0.001) level were less likely to report anxiety. Compared to current smokers, past (0.641, 0.541–0.759; *p* < 0.001) and never (0.736, 0.619–0.875; *p* < 0.001) smokers were less likely to report anxiety. A separate test of the model including the interaction between physical activity and sex was run and the interaction was not significant (*p* = 0.858).

**Table 3:** Odds ratios (OR) and 95% confidence intervals (CI) derived from binominal logistic regression analyses as indicators of cross-sectional associations between physical activity (PA) and anxiety within sex categories and the total population

	Crude^**a**^ (OR, 95%CI)			Adjusted^**b**^ (OR, 95%CI)		
	Sex		Total Population	Sex		Total Population
	Male	Female		Male	Female	
**TILDA**						
**Meeting PA Guidelines**						
No	**1**	**1**	**1**	**1**	**1**	**1**
Yes	0.842 (0.702 to 1.010)	0.951 (0.822 to 1.099)	0.853 (0.762 to 0.954)^**^	0.778 (0.624 to 0.969)	0.883 (0.742 to 1.050)	0.802 (0.702 to 0.917)^**^
**PA Dose Categories**						
Low	**1**	**1**	**1**	**1**	**1**	**1**
Moderate	0.611 (0.399 to 0.934)^*^	0.841 (0.629 to 1.125)	0.743 (0.586 to 0.943)^*^	0.590 (0.353 to 0.985)^*^	0.885 (0.635 to 1.233)	0.775 (0.588 to 1.020)
High	0.874 (0.725 to 1.053)	0.975 (0.836 to 1.137)	0.872 (0.776 to 0.980)^*^	0.802 (0.640 to 1.003)	0.883 (0.735 to 1.061)	0.807 (0.702 to 0.927)^**^
**Walking**						
Low	**1**	**1**	**1**	**1**	**1**	**1**
Moderate	0.977 (0.768 to 1.243)	1.038 (0.864 to 1.247)	1.005 (0.869 to 1.162)	0.891 (0.665 to 1.193)	1.083 (0.873 to 1.345)	0.995 (0.837 to 1.183)
High	0.911 (0.742 to 1.118)	1.036 (0.877 to 1.224)	0.944 (0.830 to 1.073)	0.920 (0.722 to 1.172)	1.003 (0.822 to 1.224)	0.972 (0.834 to 1.133)
**Mitchelstown**						
**Meeting PA Guidelines**						
No	**1**	**1**	**1**	**1**	**1**	**1**
Yes	1.007 (0.662 to 1.533)	0.786 (0.504 to 1.227)	0.828 (0.616 to 1.114)	1.069 (0.678 to 1.684)	0.753 (0.466 to 1.216)	0.918 (0.665 to 1.267)
**PA Dose Categories**						
Low	**1**	**1**	**1**	**1**	**1**	**1**
Moderate	1.452 (0.667 to 3.161)	1.186 (0.568 to 2.477)	1.247 (0.732 to 2.124)	1.859 (0.819 to 4.217)	1.024 (0.449 to 2.336)	1.286 (0.725 to 2.284)
High	0.866 (0.732 to 1.023)	0.668 (0.395 to 1.129)	0.739 (0.531 to 1.029)	0.949 (0.582 to 1.547)	0.672 (0.385 to 1.172)	0.833 (0.582 to 1.191)
**Walking**						
Low	**1**	**1**	**1**	**1**	**1**	**1**
Moderate	1.690 (1.010 to 2.829)^*^	0.488 (0.292 to 0.816)^**^	0.851 (0.595 to 1.215)	1.926 (1.111 to 3.339)^*^	0.517 (0.301 to 0.888)^*^	0.924 (0.635 to 1.344)
High	1.047 (0.626 to 1.752)	0.610 (0.363 to 1.027)	0.744 (0.519 to 1.067)	1.136 (0.659 to 1.957)	0.597 (0.338 to 1.054)	0.801 (0.545 to 1.175)
**Integrated dataset**						
**Meeting PA Guidelines**						
No	**1**	**1**	**1**	**1**	**1**	**1**
Yes	0.866 (0.732 to 1.023)	0.933 (0.813 to 1.071)	0.850 (0.765 to 0.943)**	0.843 (0.692 to 1.028)	0.871 (0.740 to 1.024)	0.865 (0.763 to 0.980)^*^
**PA Dose Categories**						
Low	**1**	**1**	**1**	**1**	**1**	**1**
Moderate	0.721 (0.497 to 1.047)	0.874 (0.666 to 1.146)	0.800 (0.644 to 0.995)^*^	0.775 (0.503 to 1.194)	0.906 (0.666 to 1.232)	0.865 (0.674 to 1.110)
High	0.886 (0.746 to 1.053)	0.946 (0.817 to 1.096)	0.858 (0.769 to 0.958)^**^	0.852 (0.696 to 1.044)	0.863 (0.726 to 1.026)	0.864 (0.758 to 0.986)^*^
**Walking**						
Low	**1**	**1**	**1**	**1**	**1**	**1**
Moderate	1.072 (0.861 to 1.335)	0.938 (0.791 to 1.113)	0.978 (0.855 to 1.119)	1.049 (0.810 to 1.358)	0.957 (0.785 to 1.167)	0.979 (0.837 to 1.145)
High	0.939 (0.776 to 1.135)	0.971 (0.829 to 1.136)	0.916 (0.812 to 1.033)	0.976 (0.783 to 1.216)	0.934 (0.776 to 1.125)	0.949 (0.824 to 1.093)

^a^Adjusted for dataset (TILDA or Mitchelstown Cohort)

^b^Adjusted for age, sex, BMI, education, smoking, marital status, dataset

^*^p < 0.05; ^**^p < 0.01

Following full adjustment, Moderate and High physical activity levels were associated with 13.5% (95% CI: − 11.0 to 32.6; *p* = 0.254) and 13.6% (95% CI: 1.4 to 24.2; *p* = 0.030) lower odds of anxiety, respectively. Following full adjustment, Moderate and High walking were associated with 2.1% (95% CI: − 14.5 to 16.3; *p* = 0.789) and 5.1% (95% CI: − 9.3 to 17.6; *p* = 0.467) lower odds of anxiety, respectively.

Anxiety symptoms were significantly lower among people meeting physical activity guidelines (5.48 ± 3.35) than those not meeting the guidelines (5.67 ± 3.51; *p* = 0.013; *g* = 0.06, 95%CI: 0.01 to 0.10). Anxiety symptoms significantly differed according to physical activity dose (*p* = 0.043). *Post-hoc* tests showed significantly lower anxiety symptoms for High (5.48 ± 3.34) compared to Low (5.67 ± 3.51, *p* < 0.001; *g* = 0.06 95%CI: 0.01 to 0.10) physical activity. There were no significant differences between High and Moderate (5.43 ± 3.39, *p* = 0.756; *g* = − 0.01, 95%CI: − 0.11 to 0.08) or Moderate and Low (*p* = 0.135; *g* = 0.07, 95%CI: − 0.02 to 0.16) physical activity. Anxiety symptoms did not significantly differ according to walking groups (*p* = 0.740).

## Discussion

This study examined the association between meeting recommended physical activity levels and anxiety among 7874 Irish adults using secondary analysis of a harmonised dataset comprised of data from TILDA and the Mitchelstown Cohort Study. Prevalence of anxiety was high compared to similar studies [62–64], potentially related to the economic downturn in Ireland in 2007 as data for the current study were collected between 2009 and 2011. Anxiety symptoms were slightly lower among adults meeting recommended physical activity guidelines and, following full adjustment for relevant covariates, meeting recommended physical activity guidelines was significantly associated with 13.5% lower odds of anxiety (HADS≥8) in the harmonized dataset, significantly associated with 19.8% lower odds of anxiety in TILDA, and non-significantly associated with 8.2% lower odds of anxiety in the Mitchelstown Cohort Study. This further highlights physical activity as an important modifiable lifestyle factor associated with anxiety symptoms and disorders along with other lifestyle factors such as sedentary behaviour [65], sleep [66], smoking [67], and alcohol use [68]; however, the magnitude of the current association was weaker than those for these lifestyle factors in previous research. Additionally, the magnitude of the present findings are consistent with cross-sectional associations between meeting recommended levels of physical activity and elevated worry symptoms [34], and smaller than previously found associations with elevated depressive symptoms among Irish adults [44, 69].

Although a dose-response between physical activity and anxiety was not supported in the current study, compared to Low physical activity, High physical activity (i.e., exceeding recommended physical activity levels) was significantly associated with lower anxiety symptoms: 13.6% lower odds of anxiety in the harmonized dataset, and 13.9% lower odds of anxiety in TILDA. The absence of a dose response is consistent with previous associations of physical activity with anxiety symptoms measured with the HADS-A [28, 33]. However, dose-responses have been observed for diagnosed generalized anxiety disorder [34] and any diagnosed anxiety disorder [36, 70], although not consistently [71].

There has been some evidence to support that, compared to physical inactivity, engaging in physical activity levels less than those recommended and increased light intensity physical activity, may convey mental health benefits [28, 37]. One accelerometry-based study demonstrated using isotemporal substitution models that substituting 30 min of light intensity physical activity for 30 min of sedentary time (while holding total wear time of the accelerometer and other activities constant) was significantly associated with a decrease in anxiety symptoms [72]. However, these findings were not supported in the present analyses of walking where anxiety symptoms and status did not differ across groups. In the Mitchelstown dataset, Moderate amounts of walking we significantly associated with 92.6% higher odds of anxiety among males, and 48.3% lower odds of anxiety among females. However, these results were potentially due to few respondents in the Moderate walking group as they were not replicated in either the TILDA or integrated datasets. The potentially important role of walking has been comparatively better-studied for depression, and somewhat remains understudied for anxiety. Given that the available literature largely supports that any physical activity is better than none, the possibility that even lower levels of moderate or vigorous intensity physical activity, increased light intensity physical activity, and increased walking may protect against anxiety and other mental health problems warrants further investigation.

Covariates that significantly contributed to the primary statistical model included age, sex, education, and smoking status, although they did not have a large influence on the magnitude of the association between physical activity and anxiety. Supporting previous findings, younger age, female sex, lower education, and smoking were significantly associated with increased likelihood of anxiety [73, 74]. From the present analyses, potentially modifiable factors such as physical activity and smoking should be considered when designing interventions, while non-modifiable factors such as age, sex, and education should be considered when identifying individuals who may be at increased risk of anxiety.

### Limitations

This study has several limitations. Due to the cross-sectional design, causality cannot be inferred from these results. Indeed, the bidirectional relationship between physical activity and anxiety has been established [63]. Unfortunately, longitudinal data could not be harmonised between the datasets. Secondly, although there are many benefits to dataset harmonisation, some limitations do exist. For example, studies may use different sampling methodologies, while differences between study variables may be difficult to reconcile in the harmonisation process. In the present study, socio-economic status could not be included as a covariate due to differences between the datasets. Finally, although a well-validated and widely used measure of physical activity, the IPAQ-SF has some limitations. For example, it only measures physical activity over the previous seven days, which may expose it to acute fluctuations in chronic physical activity behaviour, while the acute effects of physical activity on anxiety may differ from its chronic effects. Moreover, the validity of self-reported physical activity can be low, especially among low socio-economic populations [75, 76], which may predispose the results to over-reporting [77]; however, misclassification due to over-reporting would underestimate the magnitude of the physical activity-anxiety relationship, as inactive people incorrectly classified as active would be at increased risk of anxiety. Moreover, self-report measures of physical activity may be more appropriate than device measures to assess adherence to the physical activity as the guidelines were predominantly devised based on data using self-reported physical activity. Self-report physical activity measures assess the perceived time spent on physical activities but, unlike device measures, do not account for the actual fragmentation of movement throughout the activity [78]. Nonetheless, future studies would benefit from device measured physical activity which can indicate both activity duration and intensity and a stronger measure of anxiety, such as physician diagnosis. Nonetheless, this study has several notable strengths, including a large, nationally representative sample, examination of WHO physical activity recommendations, walking, and a focus on anxiety symptoms and status.

## Conclusions

After adjusting for important covariates, meeting WHO physical activity guidelines was significantly associated with 13.5% lower odds of anxiety. No apparent dose response relationship between physical activity and anxiety were observed. Additionally, walking was found to be non-significantly associated with anxiety symptoms; however, further investigation of the associations of low intensity physical activity, such as walking, and physical activity doses that do not meet recommendations are required. When considered in the context of the wider literature including prospective cohort studies and randomised controlled trials, the present findings support meeting WHO guidelines to lower odds of anxiety.

## Data Availability

The TILDA data that support the findings of this study are publicly available in the Irish Social Science Data Archive [http://www.ucd.ie/issda/data/tilda/]. The Mitchelstown data that support the findings of this study are available from Dr. Janas Harrington, University College Cork, Cork, Ireland but restrictions apply to the availability of these data, which were used under license for the current study, and so are not publicly available.

## Abbreviations

95%CI: 95% confidence intervals
BMI: Body mass index
DEDIPAC-KH: DEterminants of DIet and Physical ACtivity Knowledge Hub
GAD: Generalized anxiety disorder
HADS-A: Hospital Anxiety and Depression Scale – Anxiety subscale
IPAQ-SF: International Physical Activity Questionnaire
MET-mins: Metabolic equivalent,minutes
STROBE: Strengthening the Reporting of Observational Studies in Epidemiology
TILDA: The Irish Longitudinal Study on Ageing
WHO: World Health Organization

## References

[1] Barlow DH (2002). Anxiety and Its Disorders.

[2] World Health Organization (2017). Depression and other common mental disorders: global health estimates.

[3] Gustavsson A, Svensson M, Jacobi F (2011). Cost of disorders of the brain in Europe 2010. Eur Neuropsychopharmacol.

[4] Ormel J, Petukhova M, Chatterji S (2008). Disability and treatment of specific mental and physical disorders across the world. Br J Psychiatry.

[5] Roest AM, Zuidersma M, de Jonge P (2012). Myocardial infarction and generalised anxiety disorder: 10-year follow-up. Br J Psychiatry.

[6] Roy-Byrne PP, Davidson KW, Kessler RC (2008). Anxiety disorders and comorbid medical illness. Gen Hosp Psychiatry.

[7] Baxter AJ, Vos T, Scott KM, Ferrari AJ, Whiteford HA (2014). The global burden of anxiety disorders in 2010. Psychol Med.

[8] Luengo-Fernandez R, Leal J, Gray A (2013). Economic burden of cancer across the European Union: a population-based cost analysis. Lancet Oncol.

[9] Wilkins E, Wilson L, Wickramasinghe K (2017). European Cardiovascular Disease Statistics 2017.

[10] Frasquilho D, Matos MG, Salonna F (2015). Mental health outcomes in times of economic recession: a systematic literature review. BMC Public Health.

[11] Alonso J, Liu Z, Evans-Lacko S (2018). Treatment gap for anxiety disorders is global: results of the world mental health surveys in 21 countries. Depress Anxiety.

[12] Aggarwal NK, Balaji M, Kumar S (2014). Using consumer perspectives to inform the cultural adaptation of psychological treatments for depression: a mixed methods study from South Asia. J Affect Disord.

[13] Hidalgo RB, Tupler LA, Davidson JR (2007). An effect-size analysis of pharmacologic treatments for generalized anxiety disorder. J Psychopharmacol.

[14] Sharma T, Guski LS, Freund N, Gøtzsche PC (2016). Suicidality and aggression during antidepressant treatment: systematic review and meta-analyses based on clinical study reports. BMJ.

[15] Cuijpers P, Sijbrandij M, Koole S, Huibers M, Berking M, Andersson G (2014). Psychological treatment of generalized anxiety disorder: a meta-analysis. Clin Psychol Rev.

[16] Carpenter JK, Andrews LA, Witcraft SM, Powers MB, Smits JA, Hofmann SG (2018). Cognitive behavioral therapy for anxiety and related disorders: a meta-analysis of randomized placebo-controlled trials. Depress Anxiety.

[17] Gordon BR, McDowell CP, Lyons M, Herring MP (2017). The effects of resistance exercise training on anxiety: a meta-analysis and meta-regression analysis of randomized controlled trials. Sports Med.

[18] Herring MP, O’Connor PJ, Dishman RK (2010). The effect of exercise training on anxiety symptoms among patients: a systematic review. Arch Intern Med.

[19] Herring MP, Jacob ML, Suveg C, Dishman RK, O’Connor PJ (2012). Feasibility of exercise training for the short-term treatment of generalized anxiety disorder: a randomized controlled trial. Psychother Psychosom.

[20] Rector NA, Richter MA, Lerman B (2015). A pilot test of the additive benefits of physical exercise to CBT for OCD. Cogn Behav Ther.

[21] Gaudlitz K, Plag J, Dimeo F, Ströhle A (2015). Aerobic exercise training facilitates the effectiveness of cognitive behavioral therapy in panic disorder. Depress Anxiety.

[22] Kandola A, Hendrikse J, Lucassen PJ, Yücel M (2016). Aerobic exercise as a tool to improve hippocampal plasticity and function in humans: practical implications for mental health treatment. Front Hum Neurosci.

[23] Firth J (2018). Effect of aerobic exercise on hippocampal volume in humans: a systematic review and meta-analysis. Neuroimage.

[24] Abdallah CG (2013). A pilot study of hippocampal volume and N-acetylaspartate (NAA) as response biomarkers in riluzole-treated patients with GAD. Eur Neuropsychopharmacol.

[25] Irle E (2010). Reduced amygdalar and hippocampal size in adults with generalized social phobia. J Psychiatry Neurosci.

[26] Moylan S (2013). Exercising the worry away: how inflammation, oxidative and nitrogen stress mediates the beneficial effect of physical activity on anxiety disorder symptoms and behaviours. Neurosci Biobehav Rev.

[27] McDowell CP, Dishman RK, Gordon BR, Herring MP (2019). Physical activity and anxiety: a systematic review and meta-analysis of prospective cohort studies. Am J Prev Med.

[28] Harvey SB, Hotopf M, Øverland S, Mykletun A (2010). Physical activity and common mental disorders. Br J Psychiatry.

[29] Goodwin RD (2003). Association between physical activity and mental disorders among adults in the United States. Prev Med.

[30] World Health Organization (2010). Global recommendations on physical activity for health.

[31] Fernandez-Navarro P, Aragones MT, Ley V (2018). Leisure-time physical activity and prevalence of non-communicable pathologies and prescription medication in Spain. PLoS One.

[32] O’Loughlin EK, Low NC, Sabiston CM (2013). Symptoms of specific anxiety disorders may relate differentially to different physical activity modalities in young adults. Ment Health Phys Act.

[33] McDowell C. P., Gordon B. R., Andrews K. L., MacDonncha C., Herring M. P. (2018). Associations of physical activity with anxiety symptoms and status: results from The Irish longitudinal study on ageing. Epidemiology and Psychiatric Sciences.

[34] McDowell C, Dishman R, Vancampfort D (2018). Physical activity and generalized anxiety disorder: results from the Irish longitudinal study on ageing. Int J Epidemiol.

[35] Hallgren M, Herring MP, McDowell CP (2019). Associations of physical activity with anxiety symptoms and disorders: findings from the Swedish National March Cohort. Gen Hosp Psychiatry.

[36] Beard JR, Heathcote K, Brooks R, Earnest A, Kelly B (2007). Predictors of mental disorders and their outcome in a community based cohort. Soc Psychiatry Psychiatr Epidemiol.

[37] Kelly P, Williamson C, Niven AG, Hunter R, Mutrie N, Richards J (2018). Walking on sunshine: scoping review of the evidence for walking and mental health. Br J Sports Med.

[38] Doiron D (2013). Data harmonization and federated analysis of population-based studies: the BioSHaRE project. Emerg Themes Epidemiol.

[39] Pisani E, AbouZahr C (2010). Sharing health data: good intentions are not enough. Bull World Health Organ.

[40] Fortier I, Doiron D, Little J, Ferretti V, L’Heureux F, Stolk RP (2011). Is rigorous retrospective harmonization possible? Application of the DataSHaPER approach across 53 large studies. Int J Epidemiol.

[41] Fortier I, Raina P, Van den Heuvel ER, Griffith LE, Craig C, Saliba M (2017). Maelstrom research guidelines for rigorous retrospective data harmonization. Int J Epidemiol.

[42] Hutchinson Delyse M, Silins Edmund, Mattick Richard P, Patton George C, Fergusson David M, Hayatbakhsh Reza, Toumbourou John W, Olsson Craig A, Najman Jake M, Spry Elizabeth, Tait Robert J, Degenhardt Louisa, Swift Wendy, Butterworth Peter, Horwood L John (2015). How can data harmonisation benefit mental health research? An example of The Cannabis Cohorts Research Consortium. Australian & New Zealand Journal of Psychiatry.

[43] Von Elm E, Altman DG, Egger M, Pocock SJ, Gøtzsche PC, Vandenbroucke JP (2008). The strengthening the reporting of observational studies in epidemiology [STROBE] statement: guidelines for reporting observational studies. Gac Sanit.

[44] McDowell CP, Carlin A, Capranica L (2018). Associations of self-reported physical activity and depression in 10,000 Irish adults across harmonised datasets: a DEDIPAC-study. BMC Public Health.

[45] Brug J, van der Ploeg HP, Loyen A, Ahrens W, Allais O, Andersen LF (2017). Determinants of diet and physical activity (DEDIPAC): a summary of findings. Int J Behav Nutr Phys Act.

[46] Lakerveld J, Van Der Ploeg HP, Kroeze W, Ahrens W, Allais O, Andersen LF (2014). Towards the integration and development of a cross-European research network and infrastructure: the DEterminants of DIet and physical ACtivity (DEDIPAC) knowledge hub. Int J Behav Nutr Phys Act.

[47] Lakerveld J, Loyen A, Ling F, De Craemer M, van der Ploeg HP, O’Gorman DJ (2017). Identifying and sharing data for secondary data analysis of physical activity, sedentary behaviour and their determinants across the life course in Europe: general principles and an example from DEDIPAC. BMJ Open.

[48] Kearney PM, Cronin H, O'Regan C, Kamiya Y, Savva GM, Whelan B (2011). Cohort profile: the Irish longitudinal study on ageing. Int J Epidemiol.

[49] Kearney PM, Harrington JM, Mc Carthy VJ, Fitzgerald AP, Perry IJ (2012). Cohort profile: the Cork and Kerry diabetes and heart disease study. Int J Epidemiol.

[50] Craig CL, Marshall AL, Sjöström M, Bauman AE, Booth ML, Ainsworth BE (2003). International physical activity questionnaire: 12-country reliability and validity. Med Sci Sports Exerc.

[51] Lee PH, Macfarlane DJ, Lam TH, Stewart SM (2011). Validity of the international physical activity questionnaire short form (IPAQ-SF): a systematic review. Int J Behav Med.

[52] Zigmond AS, Snaith RP (1983). The hospital anxiety and depression scale. Acta Psychiatr Scand.

[53] Bjelland I, Dahl AA, Haug TT, Neckelmann D (2002). The validity of the hospital anxiety and depression scale: an updated literature review. J Psychosom Res.

[54] Spinhoven P (1997). A validation study of the hospital anxiety and depression scale (HADS) in different groups of Dutch subjects. Psychol Med.

[55] McDowell CP, Gordon BR, MacDonncha C, Herring MP (2019). Physical activity correlates among older adults with probable generalized anxiety disorder: results from the Irish longitudinal study on ageing. Gen Hosp Psychiatry.

[56] Vancampfort D (2018). Correlates of physical activity among community-dwelling individuals aged 65 years or older with anxiety in six low-and middle-income countries. Int Psychogeriatr.

[57] Vancampfort D, Stubbs B, Koyanagi A (2017). Physical activity correlates in people with anxiety: data from 46 low-and middle-income countries. Gen Hosp Psychiatry.

[58] World Health Organization (2000). Obesity: preventing and managing the global epidemic. Geneva.

[59] Sharpe D (2015). Your chi-square test is statistically significant: now what?. Pract Assess Res Eval.

[60] Cumming Geoff (2013). The New Statistics. Psychological Science.

[61] Cohen J (1992). A power primer. Psychol Bull.

[62] Brunes A, Gudmundsdottir SL, Augestad LB (2015). Gender-specific associations between leisure-time physical activity and symptoms of anxiety: the HUNT study. Soc Psychiatry Psychiatr Epidemiol.

[63] Da Silva MA, Singh-Manoux A, Brunner EJ (2012). Bidirectional association between physical activity and symptoms of anxiety and depression: the Whitehall II study. Eur J Epidemiol.

[64] Jonsdottir IH, Rödjer L, Hadzibajramovic E, Börjesson M, Ahlborg G (2010). A prospective study of leisure-time physical activity and mental health in Swedish health care workers and social insurance officers. Prev Med.

[65] Allen MS, Walter EE, Swann C (2018). Sedentary behaviour and risk of anxiety: a systematic review and meta-analysis. J Affect Disord.

[66] Alvaro PK, Roberts RM, Harris JK (2013). A systematic review assessing bidirectionality between sleep disturbances, anxiety, and depression. Sleep.

[67] Moylan S, Jacka FN, Pasco JA, Berk M (2012). Cigarette smoking, nicotine dependence and anxiety disorders: a systematic review of population-based, epidemiological studies. BMC Med.

[68] Carvalho AF, Stubbs B, Maes M, Solmi M, Vancampfort D, Kurdyak PA (2018). Different patterns of alcohol consumption and the incidence and persistence of depressive and anxiety symptoms among older adults in Ireland: a prospective community-based study. J Affect Disord.

[69] McDowell CP, Dishman RK, Hallgren M, MacDonncha C, Herring MP (2018). Associations of physical activity and depression: results from the Irish longitudinal study on ageing. Exp Gerontol.

[70] Ströhle A, Höfler M, Pfister H, Müller AG, Hoyer J, Wittchen HU (2007). Physical activity and prevalence and incidence of mental disorders in adolescents and young adults. Psychol Med.

[71] Ten Have M, De Graaf R, Monshouwer K (2011). Physical exercise in adults and mental health status: findings from the Netherlands mental health survey and incidence study (NEMESIS). J Psychosom Res.

[72] Dillon CB, McMahon E, O’Regan G, Perry IJ (2018). Associations between physical behaviour patterns and levels of depressive symptoms, anxiety and well-being in middle-aged adults: a cross-sectional study using isotemporal substitution models. BMJ Open.

[73] Swendsen J, Conway KP, Degenhardt L, Glantz M, Jin R, Merikangas KR (2010). Mental disorders as risk factors for substance use, abuse and dependence: results from the 10-year follow-up of the National Comorbidity Survey. Addiction.

[74] Creighton AS, Davison TE, Kissane DW (2017). The correlates of anxiety among older adults in nursing homes and other residential aged care facilities: a systematic review. Int J Geriatr Psychiatry.

[75] Dowd KP, Szeklicki R, Minetto MA (2018). A systematic literature review of reviews on techniques for physical activity measurement in adults: a DEDIPAC study. Int J Behav Med.

[76] Winckers AN, Mackenbach JD, Compernolle S, Nicolaou M, van der Ploeg HP, De Bourdeaudhuij I (2015). Educational differences in the validity of self-reported physical activity. BMC Public Health.

[77] Shook RP, Gribben NC, Hand GA (2016). Subjective estimation of physical activity using the international physical activity questionnaire varies by fitness level. J Phys Act Health.

[78] Troiano RP, McClain JJ, Brychta RJ, Chen KY (2014). Evolution of accelerometer methods for physical activity research. Br J Sports Med.

